# Intraoperative temporal interference (TI)-noninvasive brain stimulation prevents postoperative delirium in mice

**DOI:** 10.1016/j.fmre.2025.08.001

**Published:** 2025-08-16

**Authors:** Lei Liu, Ye Wang, Hui Wang, Huimin Wu, Rou Xue, Xinxin Zhang, Jingsheng Tang, Zhouwei Wang, Tingting Gu, Dan Wang, Jiannan Li, Huiming Li, Sa Wang, Jinghao Wang, Yi Zeng, Haixing Zhong, Guangchao Zhao, Hailong Dong

**Affiliations:** a Department of Anesthesiology and Perioperative Medicine, Xijing Hospital, The Fourth Military Medical University, Xi’an 710032, China; b Key Laboratory of Anesthesiology (The Fourth Military Medical University), Ministry of Education, Xi’an 710032, China; c Shaanxi Provincial Clinical Research Center for Anesthesiology Medicine, Xi’an 710032, China; d Department of Biomedical Engineering, Southern University of Science and Technology, Shenzhen 518055, China; e Shenzhen Key Laboratory of Neuropsychiatric Modulation and Collaborative Innovation Center for Brain Science, Shenzhen Institute of Advanced Technology, Chinese Academy of Sciences, Shenzhen 518055, China

**Keywords:** Perioperative delirium (POD), Temporal interference (TI), Ai-behavioral analysis, Neural modulation, Anesthesiology

## Abstract

Postoperative delirium (POD), a common neurocognitive complication in the early postoperative period associated with high morbidity in elderly and high-risk surgical patients, faces fundamental research constraints due to the lack of quantitative rodent behavioral assessment tools, hindering therapeutic innovation. Herein, we employed a 3D-AI animal behavior analysis system to evaluate spontaneous behaviors in mice post laparotomy surgery with isoflurane anesthesia. Mice exhibited apathy, bradykinesia, inflexible motion, and abnormal behavioral transitions at the 3-day after surgery. Based on unsupervised clustering and manifold analysis, we then quantized the dynamic alteration of comprehensive behavioral phenotypes in fear conditioning test. The cued memory, cognitive flexibility, and cognitive robustness were also impaired after surgery. Furthermore, we developed a novel intraoperative temporal interference-noninvasive brain stimulation (TI-NIBS), inducing a stereo-focusing electric field targeting the dentate gyrus of dorsal hippocampus, which significantly mitigated POD symptoms by restoring hippocampal neural activity and neurotransmitter homeostasis. This study elucidates a comprehensive behavioral profile of POD symptoms and provides a therapeutic non-invasive neuro-modulatory strategy to prevent POD by restoring hippocampal function.

## Graphical abstract

**Figure:**
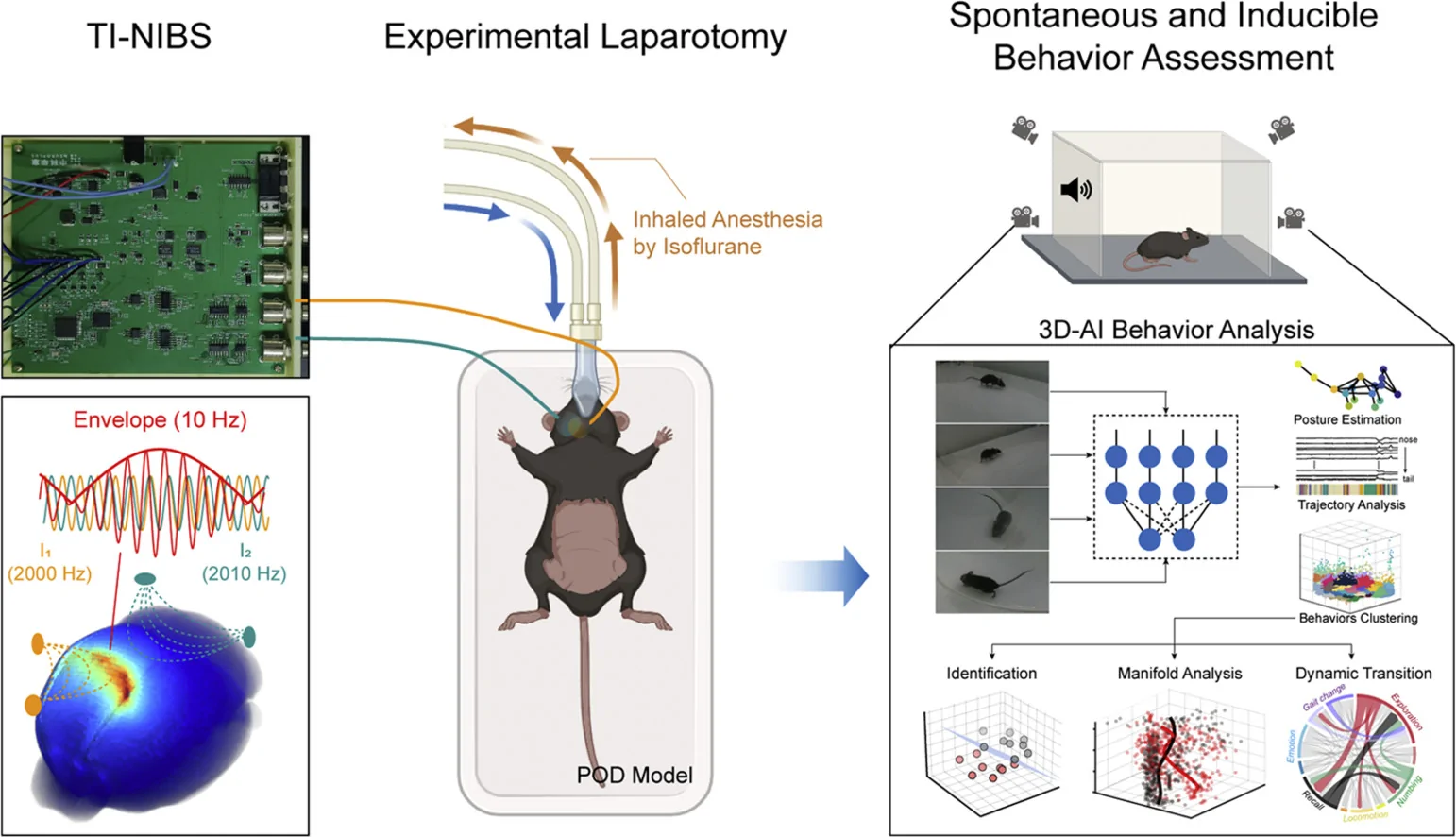

## 1 Introduction

Postoperative delirium (POD), an early neuropsychiatric symptom of perioperative neurocognitive disorders (PND), is characterized by acute cognitive decline, including memory deficits, inattention, and disorientation [1]. The incidence of POD is significantly associated with increased mortality and morbidity related to postoperative complications and prolonged hospitalization [2]. However, the mechanisms underlying POD have not yet been elucidated, thereby constraining the clinical and translational exploration of therapeutic strategies for POD.

A critical restriction in the fundamental research of POD is the absence of sensitive and robust tools for diagnosing POD in rodents. Previous studies have identified rodents with POD by evaluating their performance in several conventional behavioral paradigms, such as the open field test (OFT), elevated plus maze test (EPM), novel object recognition test, and fear conditioning test, representing a variety of cognitive functions including emotion, working memory, and contextual memory [3,4]. Owing to the low signal abundance and spatiotemporal resolution during behavioral analysis, these approaches have to oversimplify the POD phenotype. Recent advances in AI-assisted behavioral analysis (e.g., DeepLabCut, Brain Atlas) enable unsupervised, high-resolution mapping of spontaneous micro-behaviors, thereby uncovering novel mechanistic insights into POD pathogenesis [5,6]. Therefore, the application of AI-assisted fine-grained behavioral analysis to standard task-related behavior paradigms would help address the technical challenges in identifying behavioral phenotypes of POD in rodents.

In this study, we employ a 3D-AI animal behavior analysis system combined with the fear conditioning test to accurately capture the dynamic changes in spontaneous behaviors and hippocampal-dependent memory retrieval in the early period after experimental laparotomy with isoflurane general anesthesia in mice. We leveraged this multimodal platform to systematically evaluate the preventive efficacy of temporally interfering non-invasive brain stimulation (TI-NIBS) in selectively targeting the dorsal hippocampal dentate gyrus (DG) in POD. The TI-NIBS technology utilizes high-frequency electric field interference to generate amplitude-modulated low-frequency oscillations through waveform superposition, enabling frequency-selective neuronal activation that achieves precise targeting of deep brain structures typically inaccessible to conventional non-invasive stimulation methods [7,8]. This study aims to optimize the behavioral illustration of mice with POD and to provide a promising avenue for the prevention of perioperative brain complications.

## 2 Materials and methods

### 2.1 Animals

Male C57BL/6 mice aged 8 weeks (Beijing Vital River Laboratory Animal Technology Co., Ltd) were used in the experiments. All the mice were housed in a 12-hour light/12-hour dark cycle (7:00–19:00 light). The temperature and humidity were maintained at 22–24 °C and 38–42%, respectively, and free access to food and water was provided.

The design of the experiments was approved by the Ethics Committee of the Animal Experimentation Center of the Fourth Military Medical University (approval number: 250317), and the experimental procedures and details followed the principles of animal testing of the Fourth Military Medical University.

### 2.2 POD model establishment

Mice were anesthetized with 1.4% isoflurane in an anesthesia chamber for 15 min, followed by 1% isoflurane maintenance anesthesia on a heating pad at 37 °C, after which a 30-min dissection procedure was initiated. The abdominal hair was shaved, and the skin was sterilized with iodophor and incised along the midline of the abdomen from below the xiphoid process for a length of about 2 cm. This was followed by incision of the muscle and peritoneum to expose the small intestine, and about 2 cm of the small intestine was exposed from the intra-abdominal cavity to the extra-abdominal cavity. The exposed small intestine was gently massaged with the forefinger and thumb at 1-min intervals and sprayed with saline; the peritoneum, muscle, and skin were closed using a 5-0 silk suture after 10 massage sessions. Meloxicam (Mobic) cream was applied to the wounds for pain relief, and the mice were placed in a 37 °C incubator after surgery and returned to the animal house after recovery [9].

### 2.3 Temporally interfering non-invasive brain stimulation (TI-NIBS)

Based on previous studies [7,10], a mouse head model was created using computed tomography (CT) and magnetic resonance imaging (MRI) data from realistic mouse heads. The mouse cranial vault was divided into > 100 regions as potential electrode embedding sites. The finite element method and optimization framework were used to solve the multi-object optimization problem and obtain the optimal cranial-parietal electrode sites corresponding to the target stimulated brain regions. Pair 1–1: AP: 0 mm, ML: 2 mm; pair 1–2: AP: 0 mm, ML: 3 mm; pair 2–1: AP: −4 mm, ML: −1 mm; pair 2–2: AP: −8 mm, ML: 3 mm.

The pin buried at the top of the skull was connected to the output source of the stimulator, and the following stimulation parameters were set up: I_1_: 2000 Hz, 125 µA; I_2_: 2010 Hz, 125 µA. During the 30-min experiment, 10-s intervals of electrical stimulation, interspersed with 10-s rest intervals, were applied.

### 2.4 3D-AI animal behavior analysis system

This study used a multimodal behavior analysis technology to construct a complete analysis pipeline, from 2D video acquisition to 3D behavior representation [11]. During the experiment, a synchronized video acquisition system (resolution 960 × 540, frame rate 30 fps) was deployed around a circular open-field device with a diameter of 40 cm. The inner wall of the device was thoroughly cleaned with 75% ethanol before each experiment to eliminate odor interference. After the behavior recording was completed for 10 consecutive minutes, 800 frames of images were extracted for manual annotation, and the annotation information of 16 anatomical key points (including the nose, left ear, right ear, neck, left front limb, right front limb, left hind limb, right hind limb, left front claw, right front claw, left hind claw, right hind claw, back, root tail, middle tail, and tip tail) was confirmed by double-blind verification. Based on the annotated dataset, the deep convolutional neural network architecture was used for transfer learning, and the pose estimation model with spatial-temporal feature extraction ability was constructed to realize the automatic tracking of rodent body features in all the video sequences. Through the multi-view geometric correction and 3D coordinate reconstruction algorithms, the key point data of the four 2D projections were converted into a 3D skeleton model. For the reconstructed spatiotemporal motion sequence, hierarchical clustering analysis was used to deconstruct the complex behavior into 40 typical action units with clear timestamps.

Finally, the constructed multi-dimensional behavior representation matrix integrates time-domain features (millisecond-level timestamp), kinematic parameters (3D joint angle, limb length change rate), and action classification labels, and uses the Unified Manifold Approximation and Projection (UMAP) algorithm to visualize nonlinear dimensionality reduction. This technical solution provides a systematic solution for the quantitative analysis of complex behavior patterns in rodents by establishing a computational pipeline from raw video to low-dimensional behavior phenotypes.

Further hierarchical clustering of the 3D body point coordinates yielded 40 behavioral subtypes. To determine the biological significance of the 40 behaviors, we manually labeled each behavior, and each behavior was given a corresponding behavioral name, with the naming based on the Mouse Ethogram database and previous studies.

### 2.5 Memory function assessment

Mice were gently placed in the center of the context A for 180 s of environmental adaptation. Following this, they received five pairings of the conditioned stimulus (CS, 78 dB, 4.5 kHz, 30 s each) and the unconditioned stimulus (US, 0.6 mA, 2 s each); the CS was delivered at variable intervals ranging from 60 s to 180 s and each US was immediately followed by a 2-s foot shock [12,13]. The conditioned mice were returned to their home cages 60 s after the final tone. The cage was cleaned with 75% ethanol after each trial.

The contextual memory test was performed after 24 h. Mice were placed in the context A without any CS or foot shock, and the freezing time of the mice was analyzed for 10 min using the top camera that recorded 10 min of the free movement trajectory of the mice. The cage was cleaned using 75% alcohol before each experiment to eliminate odor effects. Recording and analysis were performed using the Ugo Basile Fear Conditioning System (Ugo Basile Srl, Italy).

Twenty-four hours after the initial assessment, the cued memory test was administered under ​context B, distinct from the context A​ employed previously. Before each test, the context was cleaned with 75% alcohol to eliminate odor, and then the mice were gently placed in context B. After 180 s of acclimatization, the mice were given five CS presentations (78 dB, 4.5 kHz, 30 s each, inter-tone interval: 30–120 s) without foot shock. We used five cameras to synchronously record the behavior of the mice in context B. The top-view videos were analyzed using the Ugo Basile Fear Conditioning System (Ugo Basile Srl, Italy), and the other four angles of the video were analyzed using the 3D-AI animal behavior analysis system (Bayone, Shenzhen, China).

### 2.6 Fiber photometry

In vivo calcium signal fluorescence detection was performed using a fiber optic photometric system (ThinkerTech, Nanjing, China). The excitation light intensity of the system was adjusted to 30–40 µW, the fiber optic on the head of the mouse was connected to the fiber optic photometric system, the pin was connected to the TI-NIBS stimulator, and the trigger-out of the stimulator was connected to the input of the fiber optic calcium signaling system; the baseline was recorded for 200 s after the system was connected. Then, TI-NIBS was started while recording the fiber optic photometry. The data was exported and calculated using the MATLAB software (MATLAB2024a).

### 2.7 Manifold analysis of movements

The 16 body skeleton coordinates of each frame obtained during the 30-second CS period were reduced to two dimensions by t-distributed Stochastic Neighbor Embedding (t-SNE), and the 2D data were plotted as a scatter plot (Fig. 2i). The Lempel–Ziv complexity calculation method [14] was used to perform polynomial fitting and linear fitting on the scatter plot to obtain the corresponding fitting formula. The Euclidean distance from the polynomial fitting curve corresponding to each frame to the linear fitting line was obtained to obtain 900 distance datapoints (Fig. 2j). The entropy value of this data was calculated; the higher the entropy value, the greater the distance variability between the fitting curve and the fitting line, and the lower the stability of the behavior (Fig. 2k).

$$di=\sqrt{{\left(Xpoli-Xlinei\right)}^{2}+{\left(Ypoli-Ylinei\right)}^{2}}$$

The *di* values of each sample were substituted into matlab2024a and the approximate entropy function was executed to calculate the entropy value; the core formula is as follows.

$$\Phi^{m}\left(r\right)=\frac{1}{N-m+1}\sum_{i=1}^{N-m+1}\left(\begin{array}{c} n\\ k \end{array}\right)C_{i}^{m}\left(r\right)$$

$$ApEn\left(m,r\right)=\Phi^{m}\left(r\right)-\Phi^{m+1}\left(r\right)$$

*m*: embedding dimension, which indicates the dimension that maps the time series to a high-dimensional space.

*r*: Similarity threshold (tolerance), which is usually related to the standard deviation of the data.

Φ*^m^*(*r*) and Φ*^m^*^+1^(*r*): Self-similarity statistics of the sequence pattern at dimensions m and *m* + 1, respectively.

### 2.8 Statistics

Data collection and analysis were conducted under blinded conditions to minimize experimental bias. All data are presented as mean ± standard error (SEM) or as median. Statistical analysis was conducted using Prism 10.0, MATLAB 2024a, and Origin 2018. All the data were tested for normal and chi-square distributions before analysis. For data that conformed to a normal distribution and had a chi-square variance, *t*-tests and two-way analysis of variance (ANOVA) were used for two-group and multiple group comparisons, respectively. Non-parametric tests were used for data that did not conform to normal distribution or were not variance-aligned. Results with *p* < 0.05 were considered statistically significant.

## 3 Results

### 3.1 Spontaneous behavioral changes after laparotomy surgery in mice

To precisely evaluate the differences in spontaneous behavior of mice following surgery, we employed a 3D-AI animal behavior analysis system on the third day post-modeling (Fig. 1a and b). We identified 40 distinct behavioral segments, and their feature space, generated 2D information using Uniform Manifold Approximation and Projection (UMAP) overlaying velocity information, exhibited clear clustering, with each color representing a specific behavior (Fig. 1c). Further analysis revealed that the proportions of most behavioral segments differed significantly between the groups (Fig. 1d). Dimensionality reduction and unsupervised clustering further highlighted a clear separation between the surgery and sham groups (Fig. 1e).

**Fig. 1:**
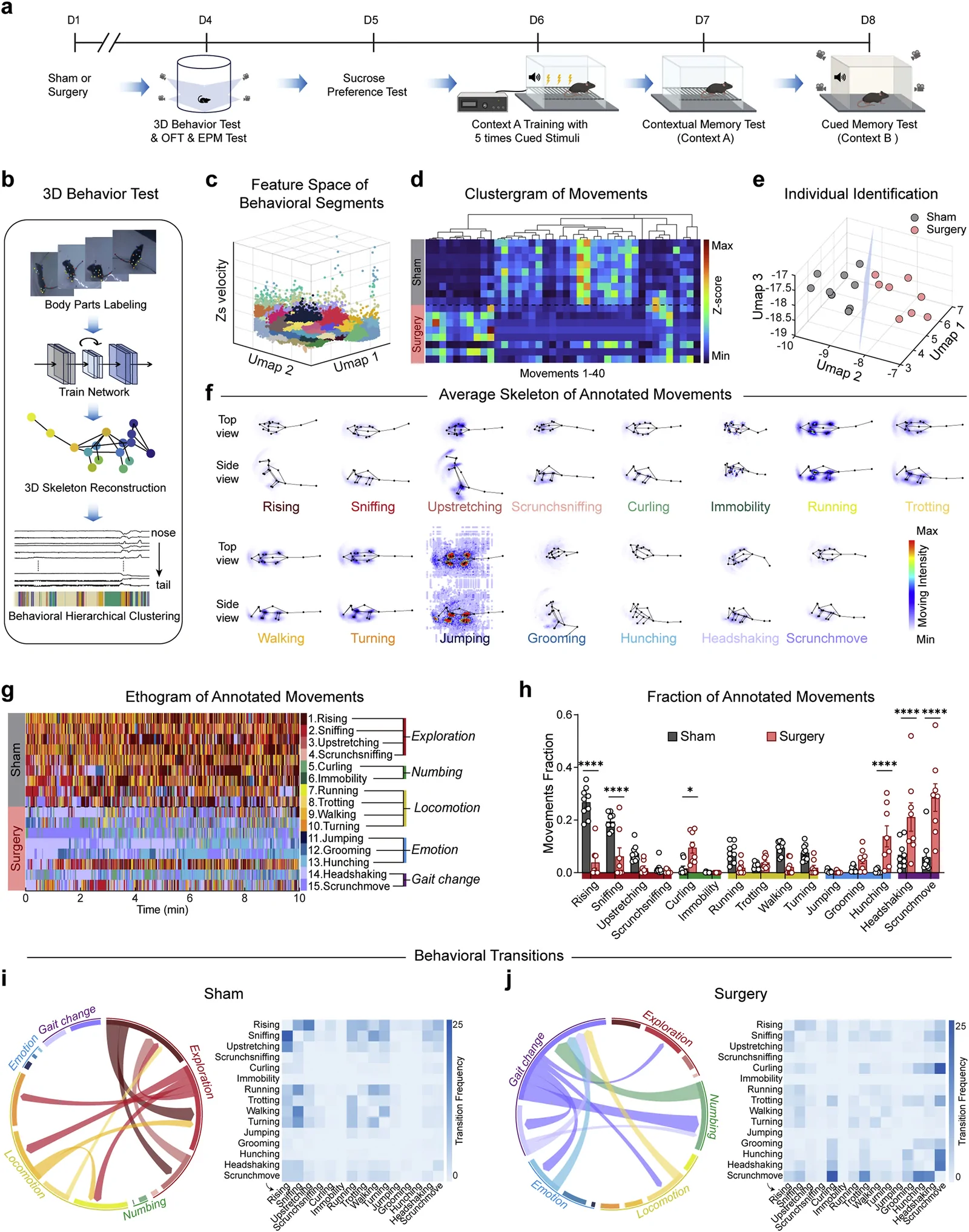
**Spontaneous behavioral changes after laparotomy surgery in mice.** (a) A schematic diagram of the experimental schedule after POD modeling by laparotomy with isoflurane general anesthesia. (b) A flow chart of the 3D-AI Animal Behavior Analysis System. (c) Spatiotemporal feature space of spontaneous phenotype segments. Different colors indicate the 40 different spontaneous movements. (d) Clustergram of samples with 40 spontaneous movements. (e) Low-dimensional individual identification for the two groups. (f) The average skeleton of all frames within each spontaneous behavior. The heatmaps indicate the movement intensity (MI) corresponding to each behavior. (g) Ethograms of the 15 annotated spontaneous behavioral movements falling into five major categories. (h) Comparison of the fraction of 15 annotated spontaneous behavioral movements between the two groups (*n* = 8–9, data are presented as mean ± SEM). Statistics: two-way ANOVA followed by Holm–Sidak post-hoc test for multiple comparisons. Rising (sham 26.76 ± 1.92% vs. surgery 3.85 ± 2.11%, *p* < 0.0001), sniffing (sham 19.41 ± 1.06% vs. surgery 6.18 ± 3.32%, *p* < 0.0001), curling (sham 1.65 ± 0.89% vs. surgery 9.53 ± 2.12%, *p* = 0.0373), hunching (sham 0.58 ± 0.24% vs. surgery 13.83 ± 4.00%, *p* < 0.0001), headshaking (sham 6.76 ± 1.73% vs. surgery: 21.12 ± 5.41%, *p* < 0.0001), and scrunch-move (sham 5.92 ± 2.28% vs. surgery 28.54 ± 5.24%, *p* < 0.0001) behaviors. (i) Left: top 10 conversion chord charts for spontaneous movements of the sham group. Right: the spontaneous movement transitions of the sham group; the previous behavioral states are listed on the Y-axis and the post states on the X-axis. (j) Left: top 10 conversion chord charts for spontaneous movements of the surgery group. Right: the spontaneous movement transitions of the surgery group; the previous behavioral states are listed on the Y-axis and the post states on the X-axis. *p < 0.05; ****p < 0.0001.

Subsequently, we performed a detailed annotation of the 40 behavior segments, categorizing them into five groups encompassing 15 distinct behaviors (Fig. 1f, Supplementary Table 1) [11]. Compared with the sham group, the rising (*p* < 0.0001) and sniffing (*p* < 0.0001) movements of the exploratory behavior in the surgery group decreased by 85% and 68%, respectively. Increased movements in the surgery group included curling (5.8-fold, *p* = 0.0373), hunching (23.8-fold, *p* < 0.0001), and headshaking (3.1-fold, *p* < 0.0001) and scrunch-move (4.8-fold, *p* < 0.0001) of numbing-, emotion-, and gait-related behaviors, respectively (Fig. 1g and h). Moreover, the speed and intensity of body movements in mice also declined after surgery (Fig. S1a). These results suggest that surgery not only impairs motor and exploratory activities but also leads to a reduction in stress- and delirium-related behaviors.

We next analyzed the distribution of the length of the behavioral segments. We found that the length of the movement sequences in the surgery group was longer than that in the sham group (0.4–0.6 segments: sham 0.1443 ± 0.007, surgery 0.0864 ± 0.0123; 0.6–0.8 segments: sham 0.1483 ± 0.0057, surgery 0.0916 ± 0.0116; >2.0 segments: sham 0.0945 ± 0.011, surgery 0.1916 ± 0.0345; Fig. S1b). The short/long preference shows that the movement sequences are longer in the surgery group than in the sham group (medians: sham 0.5186, surgery 0.2475; Fig. S1c). We also analyzed the transitions between the 15 types of behaviors and observed a substantial reduction in the frequency of transitions in the surgery group compared with the sham group (sham 697.0 ± 38.57, surgery 462.3 ± 61.87; Fig. S1d). Furthermore, in the sham group, the most frequent transitions included sequences such as rising-sniffing, upstretching-rising, and sniffing-walking. In contrast, the surgery group exhibited a distinct transition pattern, dominated by stress- and delirium-related behaviors, such as gait change-curling and gait change-head shaking (Fig. 1i and j). This suggests that surgical modeling interrupts the behavioral pattern of interchanging exploration and locomotion in normal mice and results in a pattern of locomotor transitions centered on gait changes, which are often required for transitions between other behaviors.

### 3.2 Memory deficit and behavioral instability after surgery

We used traditional behavioral methods, including the OFT, EPM, and sucrose preference test post modeling, to assess anxiety-like behavior and anhedonia in both groups. We found that the 3D-AI behavior analysis system can cover the metrics analyzed by ANY-maze software and capture more detailed activity characteristics of animals (Fig. S2). In the OFT, mice in the surgery group displayed a significant reduction in total movement distance (sham 12.79 ± 1.644 m, surgery 4.532 ± 0.818 m), alongside a decrease in the time spent in the center zone (sham 12.70 ± 2.317 s, surgery 4.125 ± 1.593 s, Fig. S3a–b). Similarly, in the EPM, the surgery group showed a marked decrease in the number of entries into the open arms (sham 14.33 ± 1.803, surgery 5.500 ± 0.906) and spent less time in the open arms (sham 44.08 ± 7.877 s, surgery 17.10 ± 3.925 s), further indicating an anxiety-like phenotype (Fig. S3c–d). Interestingly, despite these anxiety-related behaviors, the sucrose preference test revealed no statistically significant differences between the surgery and sham groups in terms of sucrose preference percentages (sham 71.14% ± 2.94%, surgery 66.39% ± 3.31%, Fig. S3e). This suggests that while the surgery group exhibited clear signs of anxiety, there was no evidence of anhedonia, as their ability to experience pleasure from sucrose remained intact.

Next, we tested the groups for contextual and cued memory using the contextual fear conditioning (CFC) test. Mice were exposed to a 30-s tone (4500 Hz, 75 dB) as the conditioned stimulus (CS), paired with a 2-s foot shock (0.6 mA) as the unconditioned stimulus (US). During the fear conditioning, the freezing time ratio was significantly lower in the surgery group than in the sham group (sham 34.18% ± 13.34%, surgery 23.21% ± 8.29%, Fig. S3f). This deficit persisted in subsequent contextual tests (sham 21.98% ± 3.711%, surgery 11.19% ± 2.441%, Fig. S3g), indicating that surgery impairs both fear memory formation and contextual memory recall.

To conduct a more refined analysis of the differences between the two groups in the cued memory test, we employed a 3D-AI animal behavior analysis system. Dimensionality reduction of 40 behavioral segments revealed distinct separation among different types of behaviors (Fig. 2a). Through manual annotation, 12 behaviors were identified and grouped into six categories. Compared with the spontaneous behavior results, an additional recall-related behavior, freezing, was identified (Fig. 2b). Detailed analysis showed a significant increase in freezing time during conditioned stimulus in the surgery group (Fig. 2c). While the freezing time during a single stimulation period was significantly lower than that in the sham group (sham 52.77% ± 4.59%, surgery 27.00% ± 2.64%, *p* < 0.0001, Fig. 2d), there was no difference between the two groups in the total freezing time during the non-stimulation period (CS-: sham 18.96% ± 1.57%, surgery 17.07% ± 1.24%, *p* = 0.7306). The total freezing time during the five-stimulus period was significantly lower in the surgery group than in the sham group (CS+: sham 52.77% ± 5.75%, surgery 27.00% ± 4.65%, *p* = 0.0006, Fig. 2e). Further, we analyzed behavioral transitions during stimulation, and in the sham group, transitions were predominantly focused on freezing, both in and out of these states. In contrast, the surgery group showed a reduction in freezing-related transitions, with an increase in transitions related to exploratory behaviors (Fig. 2f–h). To further assess behavioral stability during the stimulus period, we performed manifold analysis on the postural characteristics of the two groups. This analysis demonstrated distinct fitted curves for spatial features in the surgery and sham groups. Comparing the fitted curves to linear models and calculating the entropy values revealed significantly higher entropy in the surgery group (medians: sham 1.857, surgery 2.147, *p* = 0.0006, Fig. 2i–k). These findings support the observation of more fragmented behavioral patterns in the surgery group, potentially linked to deficits in attention and memory processing caused by surgery.

**Fig. 2:**
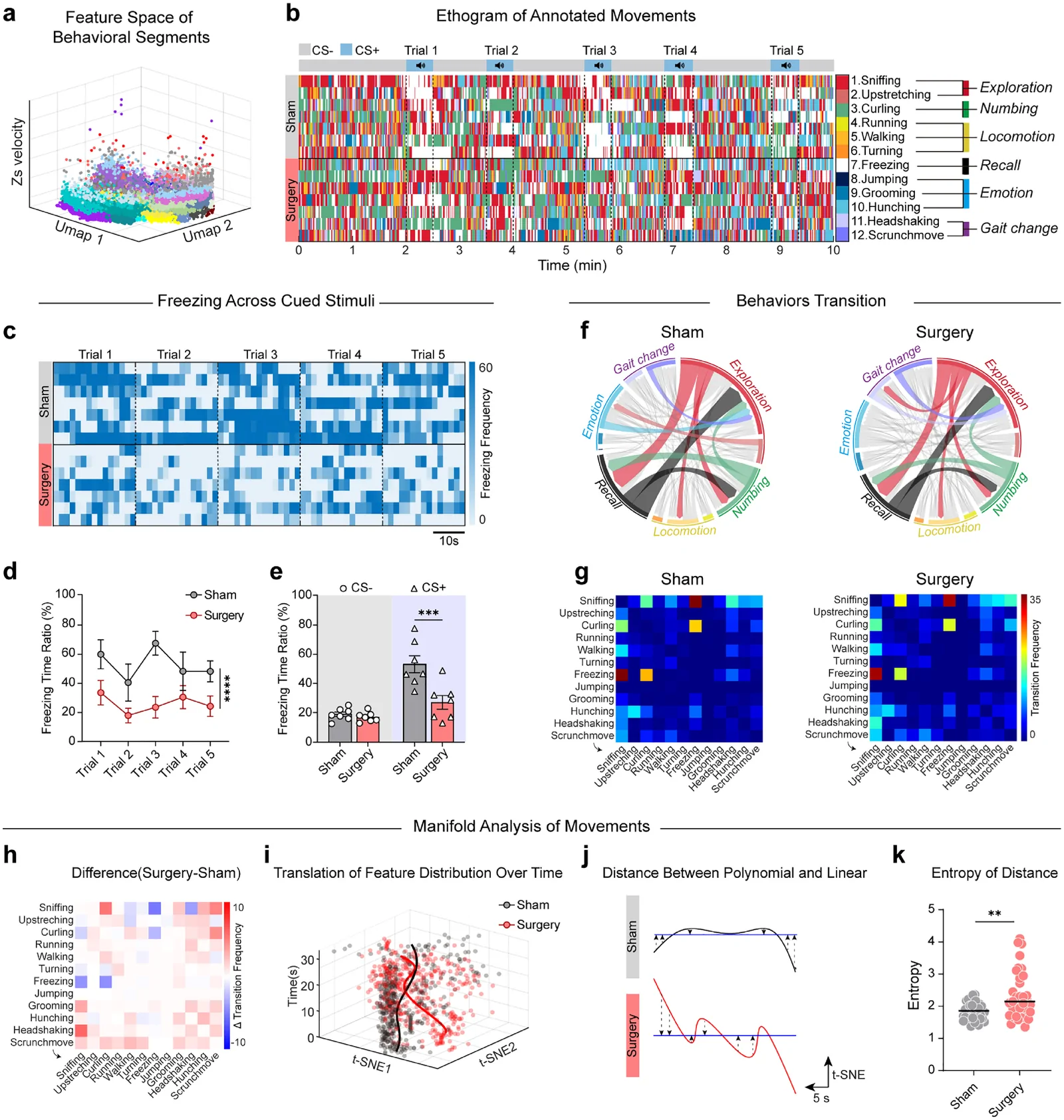
**Memory Deficit and Behavioral Instability after Surgery.** (a) Spatiotemporal feature space of predisposing phenotypes segments. (b) Top: Schematic of the cued memory test. Bottom: Ethograms of the 12 annotated predisposing behavioral movements falling into six major categories. (c) Heatmap of the number of freezing events across cued stimuli, counting the freezing event every 2 s (sampling rate = 30/s). (d) Freezing time ratio within five trials of the two groups (*n* = 7, statistics: two-way ANOVA followed by Holm–Sidak post-hoc test for multiple comparisons). (e) Freezing time ratios in the CS+ (triangle) and CS- (circle) conditions between two groups (*n* = 7, statistics: one-way ANOVA followed by Holm–Sidak post-hoc test for multiple comparisons). (f) Chordal graph for predisposing movement transitions of the two groups in the CS+ condition. Colored representations top 10 transitions. (g) The predisposing movement transitions in the CS+ condition of the two groups. The previous behavioral states are listed on the Y-axis and the post states on the X-axis. (h) Difference in predisposing movement transitions between the surgery and sham groups. (i) 3D coordinates and velocities of the body points in each frame during sound stimulation were subjected to ​t-distributed stochastic neighbor embedding (t-SNE) downscaling, and the t-SNE 2D data and time data were plotted as a 3D scatterplot; the solid line is the fitting curve of the two groups. (j) The distance between the polynomial and linear fit is shown every second, the blue line indicates the t-SNE linear fitting of each sample, the black line indicates the t-SNE polynomial fitting of each sham sample, and the red line indicates the t-SNE polynomial fitting of each surgery sample. (k) Entropy levels of the surgery and sham groups (*n* = 35, Statistics: two-sided Mann–Whitney U test). **p < 0.01, ***p < 0.001, **** *p* < 0.0001.

### 3.3 Targeted TI-NIBS selectively activates dentate gyrus of dorsal hippocampus

Grossman et al. proposed the TI-NIBS technique, which leverages the differential sensitivity of neurons to electrical stimulation—insensitivity to high-frequency stimulation but sensitivity to low-frequency stimulation. This method uses two high-frequency electrical stimuli with a frequency difference to create a low-frequency envelope within the stimulation region, enabling neural modulation (Fig. 3a). Building on this theory, we developed a TI-NIBS stimulator capable of generating consistent high-frequency currents across different loads by employing two voltage-controlled current sources and a 1:1 transformer (Fig. 3b). During the experiments, CH1 and CH2 were connected to the cranial vertex of the mouse, with a universal asynchronous receiver/transmitter (UART) interface linking to a control computer. The Trigger Out and Trigger In interfaces enable synchronized operation with other devices (Fig. 3c).

**Fig. 3:**
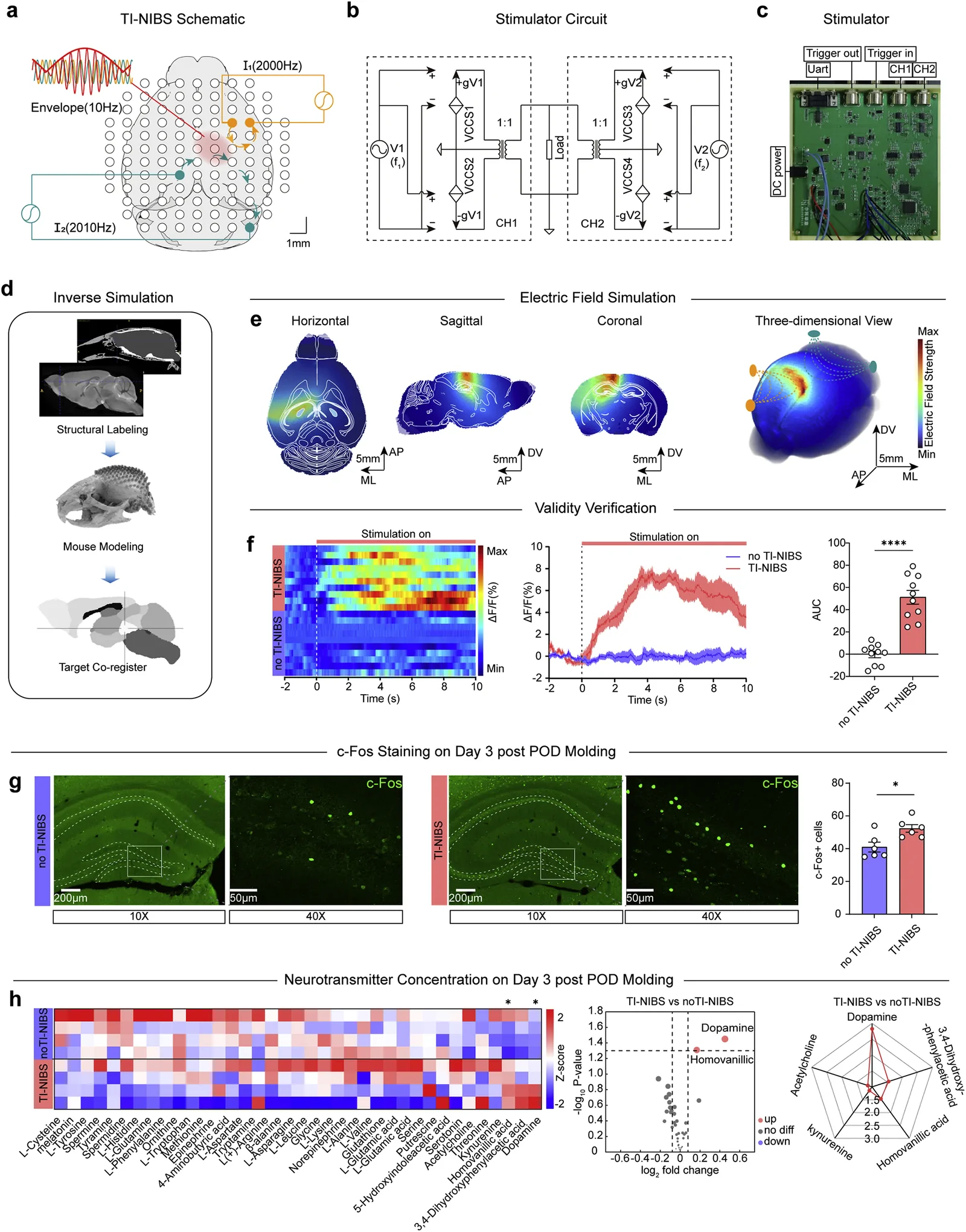
**Targeted TI-NIBS selectively activates the dentate gyrus of the dorsal hippocampus.** (a) Schematic diagram of the TI-NIBS procedure: the orange and cyan dots indicate the first and second pair of electrodes adding 2000 Hz and 2010 Hz high-frequency sinusoidal AC currents, respectively. A low-frequency envelope of 10 Hz is created between the two high-frequency currents. (b) The circuit comprises two identical channels (CH1/CH2). The voltage waveform (V1) of each channel is amplified by an operational amplifier to drive two voltage-controlled current sources: voltage-controlled current source 1 (VCCS1) generates in-phase current, while VCCS2 produces an inverted current. A 1:1 isolation transformer replicates the constant current from the sources to the load, maintaining a current-to-voltage ratio 'g'. (c) Stimulator entity. CH1 and CH2 are connected to the pin on the head of the mouse, the UART is connected to the control software, the trigger out and trigger in are the output and input of the marker, and DC power is used to connect to the DC power supply. (d) Flowcharts for inverse simulation. (e) Reverse-simulated electric field heatmaps of the dorsal hippocampus in the horizontal, sagittal, and coronal planes. The simulated electric field is generated by two pairs of electrodes in the mouse brain. (f) Fiber photometry measuring calcium signals responding to the TI-NIBS and no-TI-NIBS sides of the hippocampus (*n* = 10, statistics: two-sided Mann–Whitney U test). (g) c-Fos Immunofluorescence staining in the DG region of the hippocampus of the TI-NIBS and no-TI-NIBS groups (*n* = 6, statistics: two-sided paired *t*-test). (h) The heatmap shows the neurotransmitter levels in the study groups. * Represents *p* < 0.05 between the TI-NIBS and no-TI-NIBS groups. In the volcano plots, red and blue represent relatively increased and decreased expression levels, respectively. Radar charts for the five neurotransmitters that are increased in the TI-NIBS group compared with the no-TI-NIBS group. *p < 0.05, ****p < 0.0001.

To enhance the precision of targeted stimulation sites, we developed a reverse modeling technique for electrode placement. We used CT and MRI data from mouse heads to label and extract relevant tissue information. Potential electrode positions were identified based on the accessible surgical range of the cranial surface. These positions were then integrated with the head model data to construct a detailed 3D model of the mouse head. We employed the finite element method (FEM) to simulate the electric fields generated by transcranial electrical stimulation to identify the optimal electrode placement targeting the dentate gyrus (DG) area (Fig. 3d and e). The effectiveness of this targeting was then validated through c-Fos immunofluorescence staining and in vivo calcium signal recordings. After 90 min of TI-NIBS intervention, the number of c-Fos-positive cells in the stimulated side was significantly higher than that in the non-stimulated side (Fig. S4a and b). In vivo fiber optic calcium signal recording during TI-NIBS stimulation also found that the calcium signal intensity of the stimulated side was significantly higher than that of the non-stimulated side (medians: TI-NIBS 51.34, no TI-NIBS 1.699, *p* < 0.0001, Fig. 3f). In order to explore the effect of TI-NIBS stimulation alone on hippocampal neurons in mice for a long time, we measured the expression of c-Fos and the neurotransmitter levels in the hippocampus after 3 days in the stimulated and non-stimulated mice. It was found that the c-Fos content in the hippocampus in the TI-NIBS group was higher than that in the no TI-NIBS group after 3 days (TI-NIBS 52.33 ± 2.33, no TI-NIBS 41.00 ± 3.01, *p* = 0.0139, Fig. 3*g*), indicating that the regulatory effect of TI-NIBS on neurons could last for at least 3 days. We also found that the dopamine and homovanillic acid concentrations in the hippocampus changed significantly, and the levels were higher in the TI-NIBS group than in the non-stimulation group (dopamine: TI-NIBS 0.045 ± 0.010, no TI-NIBS 0.016 ± 0.002, *p* = 0.035; homovanillic acid: TI-NIBS 0.072 ± 0.002, no TI-NIBS 0.049 ± 0.009, *p* = 0.049, Fig. 3h).

### 3.4 Intraoperative TI-NIBS ameliorates abnormal spontaneous behavioral phenotypes in mice after surgery

To evaluate the potential therapeutic effects of TI-NIBS on spontaneous behavioral deficits induced by surgery, stimulation electrodes were implanted in mice one week prior to the procedure. TI-NIBS (10 Hz, 125 µA) was applied during surgery. Starting on the third day post-surgery, a comprehensive series of behavioral tests was conducted, including the 3D-AI animal behavior analysis system, OFT, EPM, sucrose preference tests, and fear conditioning (Fig. 4a).

**Fig. 4:**
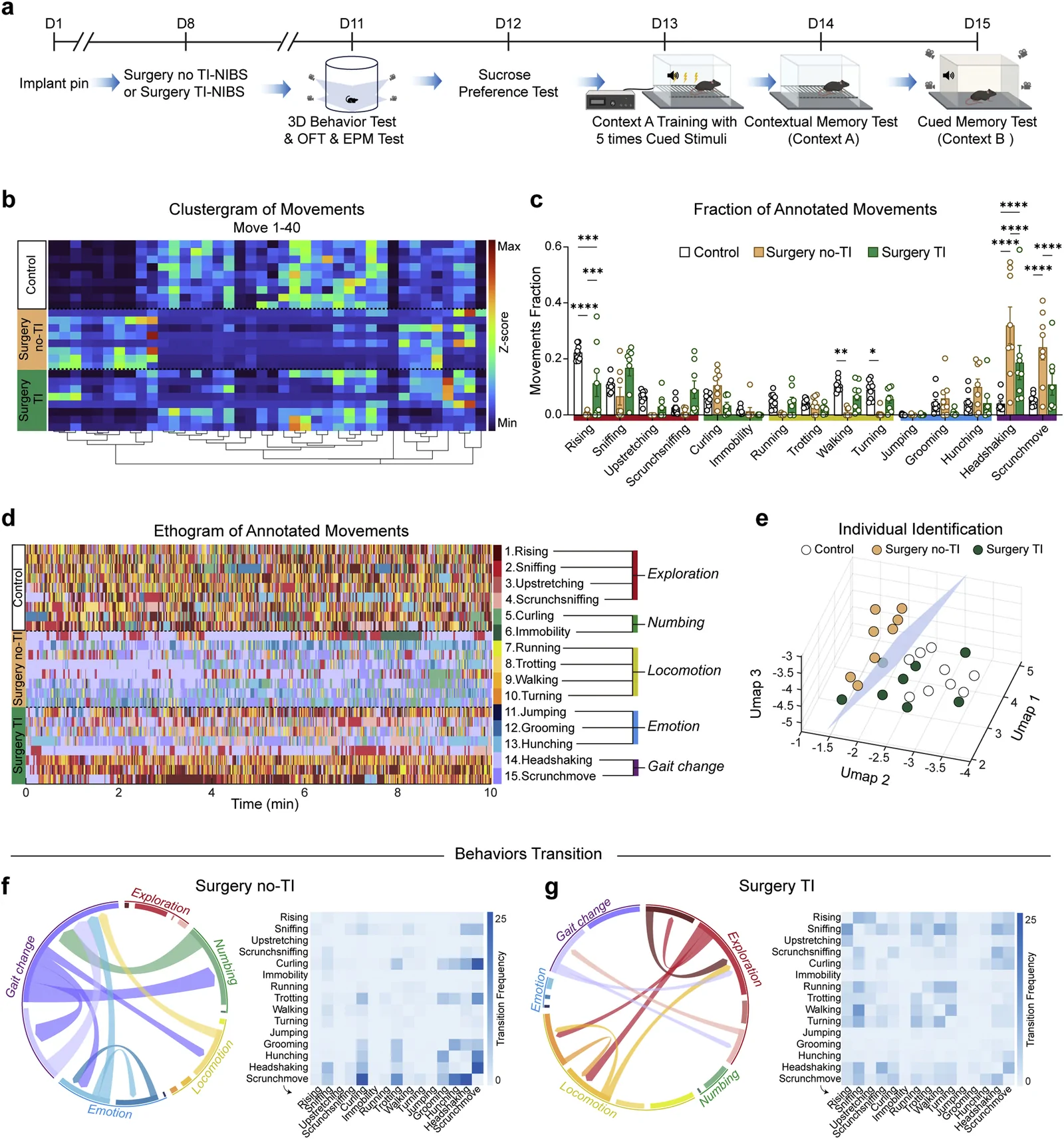
**Intraoperative TI-NIBS ameliorates abnormal spontaneous behavioral phenotypes in mice after surgery.** (a) Schematic diagram of behavioral testing after TI-NIBS. (b) Clustergram of samples with 40 spontaneous phenotypes. (c) The fraction of 15 annotated spontaneous behavioral movements was compared among three groups (*n* = 8–9; Statistics: two-way ANOVA followed by the Holm–Sidak post-hoc test for multiple comparisons). Rising (control 0.226 ± 0.009, surgery no-TI 0.004 ± 0.002, surgery TI 0.116 ± 0.048; control vs. surgery no-TI *p* < 0.0001, surgery no-TI vs. surgery TI *p* = 0.0006, control vs. surgery TI *p* = 0.0006), head shaking (control 0.042 ± 0.010, surgery no-TI 0.324 ± 0.067, surgery TI 0.190 ± 0.062; control vs. surgery no-TI *p* < 0.0001, surgery no-TI vs. surgery TI *p* < 0.0001, control vs. surgery TI *p* < 0.0001), scrunch move (control 0.055 ± 0.007, surgery no-TI 0.245 ± 0.046, surgery TI 0.111 ± 0.039; control vs. surgery no-TI *p* < 0.0001, surgery no-TI vs. surgery TI *p* < 0.0001, control vs. surgery TI *p* = 0.064), walking (control 0.109 ± 0.008, surgery no-TI 0.010 ± 0.067, surgery TI 0.071 ± 0.017; control vs. surgery no-TI *p* = 0.003, surgery no-TI vs. surgery TI *p* = 0.098), and turning (control 0.092 ± 0.012, surgery no-TI 0.007 ± 0.005, surgery TI 0.071 ± 0.018; control vs. surgery no-TI *p* = 0.0144, surgery no-TI vs. surgery TI *p* = 0.187) behaviors. (d) Ethograms of the 15 annotated spontaneous behavioral movements falling into five major categories. (e) Low-dimensional representation of the three animal groups. (f) Left: top ten conversion chord charts of spontaneous movement transitions of the surgery no-TI group. Right: the spontaneous movement transitions of the surgery no-TI group; the previous behavioral states are listed on the Y-axis and the post states on the X-axis. (g) Left: top ten conversion chord charts of spontaneous movement transitions of the surgery TI group. Right: the spontaneous movement transitions of the surgery TI group; the previous behavioral states are listed on the Y-axis and the post states on the X-axis. *p < 0.05, **p < 0.01, ***p < 0.001, ****p < 0.0001.

After TI, we observed changes in 38 kinematic parameters related to spontaneous behavior (Fig. S5a). A significant increase in exercise intensity was found in the intraoperative TI-NIBS group. We observed significant changes in 40 movements following TI stimulation in the surgery group (Fig. 4b). Intraoperative TI-NIBS can increase the proportion of rising behavior after laparotomy exploration 29 times, reaching 51% of the control group. It can also reduce the head shaking (41%) and scrunch move (55%) gait change behaviors that increase after laparotomy. There were no statistically significant differences in improvements in walking and turning under reduced locomotion behaviors after exploratory laparotomy.

Intraoperative TI-NIBS intervention demonstrated significant behavioral modulation following exploratory laparotomy. The surgery TI group exhibited a 29-fold increase in rising behavior compared with the surgery no-TI group, attaining 51% of the control group baseline level. Concurrently, TI-NIBS attenuated laparotomy-induced gait change, manifesting as 41%- and 55%-reductions in head shaking and scrunch movement frequencies, respectively. However, quantitative analysis indicated that intraoperative TI-NIBS does not result in significant improvement in locomotion behavior; there was no statistically significant difference in the proportion of walking turning movements compared with the surgery group (Fig. 4c).

Furthermore, intraoperative TI-NIBS reversed the prolongation of movement segments caused by exploratory laparotomy (0.6–0.8 segments: control 0.12 ± 0.012, surgery no-TI 0.083 ± 0.007, surgery TI 0.13 ± 0.017; >2.0 segments: control 0.12 ± 0.016, surgery no-TI 0.23 ± 0.018, surgery TI 0.15 ± 0.034; Fig. S5b). The short/long preference showed that the surgery no TI-NIBS group (0.078 ± 0.063) was significantly lower than that of the control group (0.398 ± 0.076), but was not significantly different from the surgery TI-NIBS group (0.319 ± 0.105; Fig. S5c). The surgery no-TI-NIBS group showed a lower number of transitions between behaviors than other groups (control 508.8 ± 34.86, surgery no-TI 316.6 ± 34.17, surgery TI 478.9 ± 63.14, Fig. S5d). Significant group differences were also observed in the transitions between 15 categorized movements.

In the postoperative stages, the surgery no-TI group still showed a behavioral pattern dominated by gait changes, mood-related changes, and rigidity, whereas exploratory movement and some gait changes were the main behavioral patterns in the TI group (Fig. 4f and g). This suggests that intraoperative TI can ameliorate the surgery-related changes in motor patterns in spontaneous behavior.

### 3.5 Intraoperative TI-NIBS improves neurocognition after surgery

To investigate the effects of TI-NIBS on cognition and emotional behavior in mice post-surgery, several behavioral assays were conducted on different days following the procedure (Fig. 4a). We observed that TI-NIBS can alleviate anxiety-like behaviors induced by surgery, improved overall travel distance (control 13.26 ± 1.126, surgery no-TI 2.609 ± 0.4772, surgery TI 6.357 ± 1.234), reversed the time spent in the central area (control 12.70 ± 1.158, surgery no-TI 3.70 ± 1.57, surgery TI 12.65 ± 1.15), improved the number of entries into the open arm and time spent in it (number: control 14.78 ± 1.321, surgery no-TI 5.375 ± 1.194, surgery TI 11.00 ± 1.637; time spent: control 43.88 ± 3.672, surgery no-TI 20.00 ± 3.426, surgery TI 35.79 ± 5.952); however, it had no effect on anhedonia-like behavior (control 74.95 ± 4.40, surgery no-TI 74.42 ± 4.47, surgery TI 73.90 ± 4.03, Fig. S6a–e). Besides, in the fear conditioning paradigm, TI-NIBS-treated mice displayed a higher percentage freezing time that was close to the level in the control group during fear conditioning (control 34.98 ± 12.44, surgery no-TI 19.93 ± 7.179, surgery TI 41.98 ± 12.73); however, no significant intervention-related changes were found in contextual memory (control 31.39 ± 5.046, surgery no-TI 16.08 ± 4.509, surgery TI 40.73 ± 9.088, Fig. S6f–g), suggesting that TI-NIBS can ameliorate the memory deficits following surgery. At the same time, we performed a novel object recognition test after laparotomy and after intraoperative TI-NIBS intervention. There was no statistically significant difference in the proportion of mice exploring novel objects between the three groups (control 63.27 ± 4.07, surgery no-TI 60.83 ± 6.51, surgery TI 55.35 ± 5.77, Fig. S6h).

Additionally, the 3D-AI animal behavior analysis system revealed significantly different ethograms of annotated movements (Fig. 5a). Mice in the surgery TI group exhibited lower freezing time during the cued memory retrieval (Fig. 5b–d). Detailed analysis showed a significant decrease in freezing time during conditioned stimulus in the surgery TI group (Fig. 5b). The freezing time, which was close to that of the sham group, during a single stimulation period was significantly higher than that of the surgery no-TI group (control 60.73 ± 2.01, surgery no-TI 22.00 ± 1.27, surgery TI 38.29 ± 2.30; control vs. surgery no-TI, *p* < 0.0001; surgery no-TI vs. surgery TI, *p* = 0.027; control vs. surgery TI, *p* = 0.0005; Fig. 5c), while there was no difference between the two groups in the total freezing time during the non-stimulation period (control 23.50 ± 2.53, surgery no-TI 13.17 ± 2.56, surgery TI 18.97 ± 3.41; control vs. surgery no-TI, *p* = 0.42; surgery no-TI vs. surgery TI, *p* = 0.68; control vs. surgery TI, *p* = 0.91). The total freezing time during the five-stimulus period was significantly higher than that of the surgery no-TI group (control 60.73 ± 5.10, surgery no-TI 22.00 ± 3.64, surgery TI 40.82 ± 5.89; control vs. surgery no-TI, *p* < 0.0001; surgery no-TI vs. surgery TI, *p* = 0.0071; control vs. surgery TI, *p* = 0.0065, Fig. 5d). Analysis of behavioral transitions during the stimulus phase revealed that the surgery no TI-NIBS group exhibited more frequent transitions between movements (Fig. 5e–g). The manifold analysis found that TI-NIBS reversed the surgery-related abnormal increase in entropy (control 1.76 ± 0.11, surgery no-TI 2.35 ± 0.13, surgery TI 1.92 ± 0.079; control vs. surgery no-TI, *p* = 0.0004; surgery no-TI vs. surgery TI, *p* = 0.011; control vs. surgery TI, *p* = 0.27; Fig. 5h–j).

**Fig. 5:**
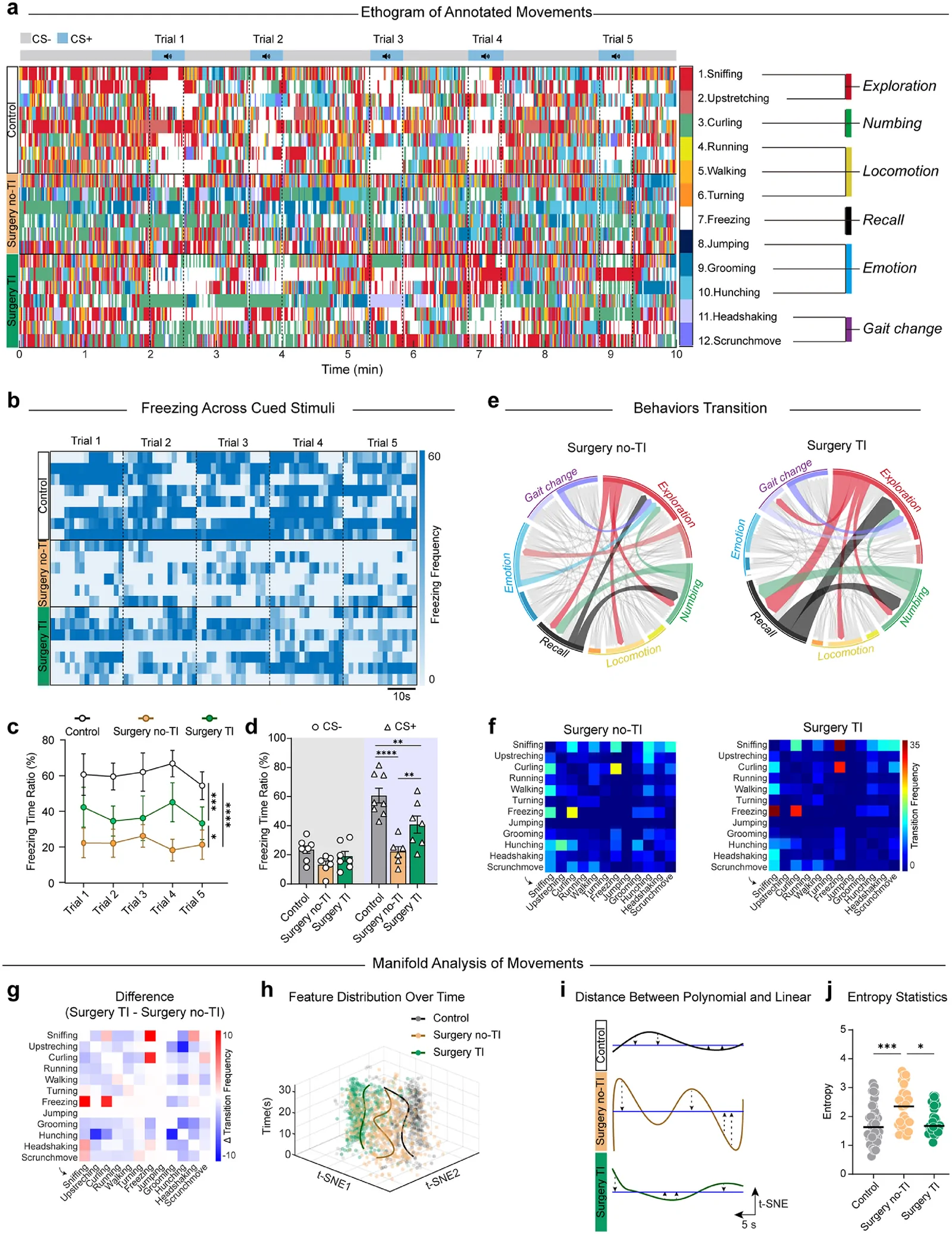
**Intraoperative TI-NIBS improves neurocognition after surgery.** (a) Top: Schematic representation of the cued memory test. Down: Ethograms of the 12 annotated predisposing behavioral movements falling into six major categories. (b) Heatmap of the number of freezing events across cued stimuli, counting the freezing events every 2 s (sampling rate = 30/s). (c) Line plot of freezing time ratio within five trials in the three groups of animals (*n* = 6–8, Statistics: two-way ANOVA followed by the Holm–Sidak post-hoc test for multiple comparisons). (d) Freezing time ratio in the CS+ (triangle) and CS- (circle) conditions (*n* = 6–8, Statistics: one-way ANOVA followed by the Holm–Sidak post-hoc test for multiple comparisons). (e) Chordal graph of predisposing movement transitions of the surgery no-TI and surgery TI groups. Colored representations indicate the top ten transitions. (f) The predisposing movements transitions of the surgery no-TI and surgery TI groups. The previous behavioral states are listed on the Y-axis and the post states on the X-axis. (g) Difference in the behavioral movement transitions of the surgery no-TI and surgery TI groups. (h) 3D coordinates and velocities of the body points in each frame during sound stimulation were subjected to t-SNE downscaling, and the t-SNE 2D data and time data were plotted as a 3D scatterplot. (i) The distance between the polynomial and linear fitting is shown every second, the blue line indicates the t-SNE linear fitting of each sample, the yellow line indicates the t-SNE polynomial fitting of each surgery no-TI sample, and the green line indicates the t-SNE polynomial fitting of each surgery TI sample. (j) Levels of the entropy of the three groups (Statistics: two-way ANOVA followed by the Holm–Sidak post-hoc test for multiple comparisons). * *p* < 0.05, ** *p* < 0.01, *** *p* < 0.001, **** *p* < 0.0001.

### 3.6 TI-NIBS restores neuronal activity and neurotransmitter system after surgery

We performed c-Fos immunofluorescence staining on four groups of mice three days after modeling. The results showed a significant reduction in c-Fos expression in the DG of the hippocampus in the surgery group compared with that in the sham group (sham 40 ± 2.16, surgery 30 ± 2.71, *p* = 0.016); this decrease could be reversed by TI-NIBS (surgery no-TI 31.5 ± 0.99, surgery TI 48 ± 1.71, *p* < 0.0001, Fig. 6a–b). We also examined the in vivo fiber calcium signal intensity in the dorsal hippocampus during acoustic stimulus-induced memory, and found that while the calcium signal intensity was reduced in mice after laparotomy (sham 0.168 ± 0.063, surgery −0.008 ± 0.051, *p* = 0.043), intraoperative TI-NIBS intervention was effective in increasing the reduced calcium signal (surgery no-TI −0.035 ± 0.051, surgery TI 0.21 ± 0.025, *p* = 0.0004, Fig. 6c and d). These findings suggest a potential mechanism for TI-NIBS to prevent POD-related behaviors.

**Fig. 6:**
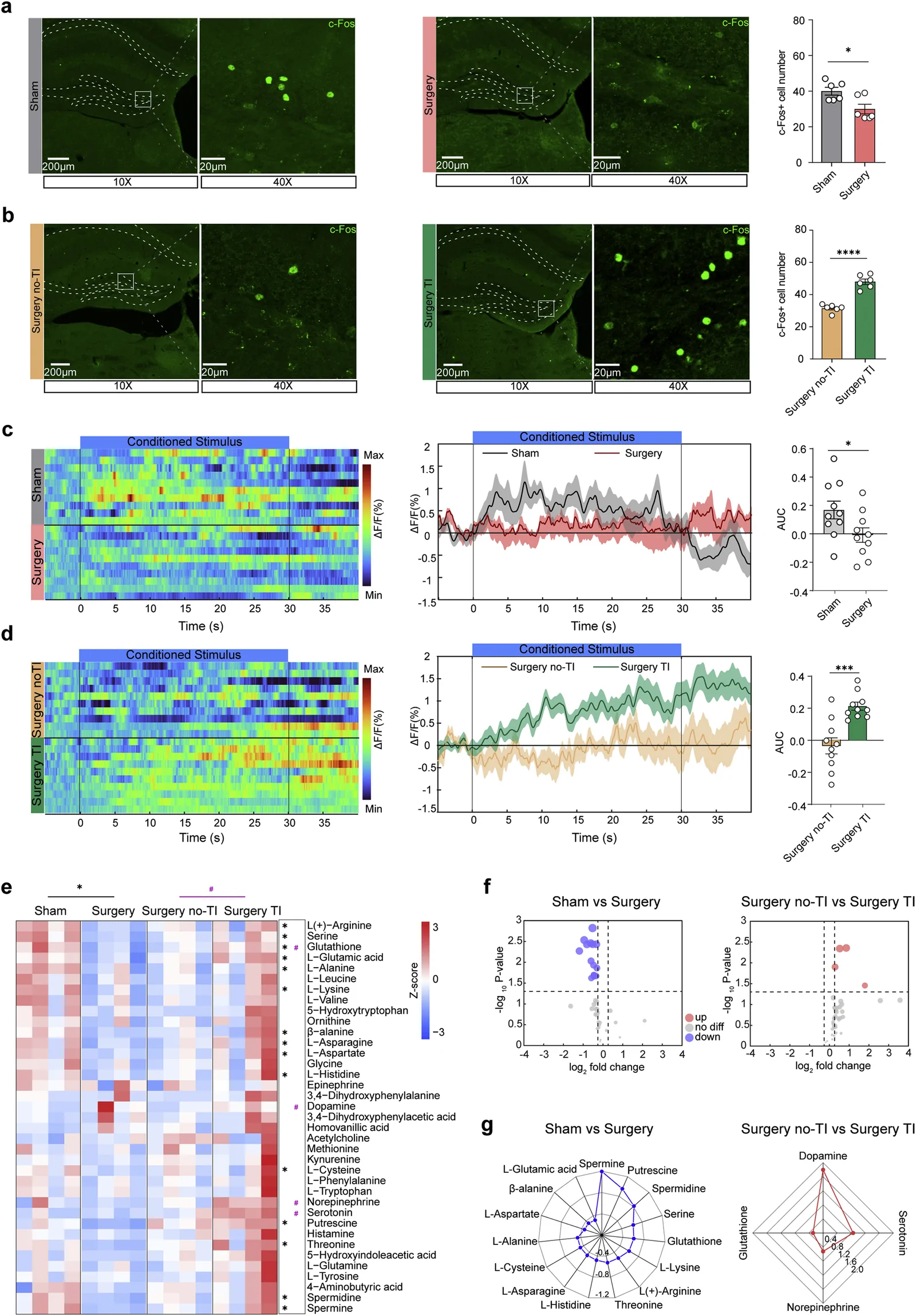
**TI-NIBS restores neuronal activity and the neurotransmitter system after surgery.** (a and b) c-Fos immunofluorescence staining in the DG region: (a) Sham and surgery groups, (b) Surgery no-TI and surgery TI groups (*n* = 6, statistics: two-sided unpaired *t*-test). (c–d) In vivo fiber recording in the DG region: (c) Sham and surgery groups, (d) surgery no-TI and surgery TI groups (*n* = 10, statistics: two-sided unpaired *t*-test). (e) Heatmap showing the neurotransmitter levels in the four groups. * Represents *p* < 0.05 between the sham and surgery groups, # represents *p* < 0.05 between the surgery no-TI and surgery TI groups. (f) Volcano plots for sham vs. surgery and surgery no-TI vs. surgery TI group comparisons. Red and blue represent the relatively increased and decreased expression levels. (g) Left: Radar charts for the 15 neurotransmitters that are significantly lower in the surgery group than in the sham group. Right: Radar charts for the four neurotransmitters that are significantly elevated in the surgery TI group compared with the surgery no-TI group. *p < 0.05, ***p < 0.001, ****p < 0.0001.

We also conducted hippocampal neurotransmitter analysis to compare neurotransmitter levels in the four groups three days after modeling. Compared with the sham group, 15 neurotransmitters were significantly reduced in the surgery model group, among which spermidine and putrescine showed the most significant changes. Notably, in the group that underwent TI-NIBS, four neurotransmitters showed elevated levels, with dopamine exhibiting the most pronounced increase (Fig. 6e and f). Of these, glutamine decreased after surgical modeling and increased significantly after TI-NIBS (Fig. 6g).

## 4 Discussion

In the current study, we combined 3D-AI animal behavior analysis with the fear conditioning test to evaluate the variation in spontaneous behaviors under the default situation, and inducible cognitive behaviors in mice that underwent laparotomy with isoflurane general anesthesia. We observed that surgery led to impaired behavioral performance, such as apathy, altered behavior transitions, motional bradykinesia, and hypo-flexibility, as well as memory deficit and cognitive instability. Notably, DG-targeted TI-NIBS improved spontaneous behavioral phenotypes and alleviated neurocognitive and memory deficits, possibly by restoring neuronal activity and normalizing neurotransmitter systems in the DG of the dorsal hippocampus. This phenomenon suggests that TI-NIBS may help prevent POD development.

POD is characterized by inattention, altered level of consciousness, and acute changes in cognitive function [15]. However, the heterogeneity of phenotypes in patients with POD gives rise to a severe complication in individual identification, which significantly hinders the innovation of preventive and therapeutic strategies for POD [16,17]. This challenge also extends to the fundamental research of perioperative neurocognitive disorders. For instance, scientists utilized the novel object test, Morris water maze, Barnes maze, fear conditioning test, and other standard behavioral paradigms to detect long-term cognitive deficits such as postoperative cognitive dysfunction after surgery [3,18,19]. However, POD symptoms cannot be simply described as a short-term cognitive deficit. Recent efforts to address these challenges include using the 3D-AI animal behavior analysis system to monitor postoperative emotional and cognitive changes [20]. However, this approach fails to capture other important behavioral aspects, particularly in the cognitive instability and flexibility during task execution. In our study, by virtue of this combination, the 3D-AI animal behavior analysis system revealed a significant decline in behavioral flexibility, as well as memory deficit and behavioral instability. These findings emphasize the complex, multifactorial nature of POD and the need for comprehensive methods to assess the full spectrum of behavioral phenotypes.

Current approaches to POD prevention and treatment include preoperative, intraoperative, and postoperative interventions [15], such as minimizing polypharmacy [21], enhanced recovery after surgery (ERAS) approach, multimodal analgesia, non-steroidal anti-inflammatory drugs (NSAIDs), and low-dose dexmedetomidine. These approaches primarily mitigate high-risk perioperative triggers to achieve therapeutic goals. Pharmacological interventions focus on neuroinflammatory pathways through suppression of pro-inflammatory cytokines (e.g., IL-6, TNF-α) or cyclooxygenase (COX) activity inhibition. Dexmedetomidine, a selective α2-adrenergic receptor agonist, additionally suppresses central sympathetic outflow by reducing norepinephrine release, thereby attenuating surgical stress responses [22]. TI-NIBS achieves precise targeting of deep brain structures with millimeter-level spatial resolution by leveraging waveform superposition to generate frequency-selective neuronal activation, thereby overcoming the spatial limitations of conventional non-invasive neuromodulation to directly modulate neuronal excitability within emotion- and cognition-related circuits (e.g., prefrontal-limbic pathways) [23]. In contrast, traditional electrical stimulation methods such as transcranial direct/alternating current stimulation (tDCS/tACS) induce diffuse, non-selective cortical activation, often failing to engage subcortical nuclei (e.g., thalamocortical or limbic structures) while risking unintended interference with adjacent neural networks or inadequate deep-brain penetration [24]. The combined application of TI-NIBS with other therapeutic modalities could potentially enhance treatment efficacy and improve pharmacological sensitivity to therapeutic agents.

Despite its promise, clinical translation of TI-NIBS faces critical challenges. In this study, we determined hippocampal stimulation targets through inverse modeling based on prior CT and MRI data from realistic mouse heads. However, translating this approach to humans requires individualized MRI-guided electric field simulations to account for anatomical variability, which may limit accessibility in resource-limited clinical settings. Additionally, optimal stimulation parameters (e.g., frequency, intensity) and timing (pre- vs. postoperative administration) remain to be determined. Furthermore, although spatial precision offered by TI-NIBS may theoretically surpass that of conventional methods like tDCS/tACS in both efficacy and safety, direct comparative clinical evidence is lacking. Multicenter trials are urgently needed to validate its advantages in delirium prevention and cognitive recovery.

By comparing neuronal responses between the stimulated and non-stimulated sides of the hippocampus, we confirmed that TI-NIBS could accurately target the dorsal hippocampal DG region. Mechanistic studies on learning and memory suggest that the DG contributes to the formation and consolidation of episodic memories through regulation in engram cell encoding [[25], [26], [27]]. Its interaction with other brain regions, like the amygdala, also modulates emotional states such as anxiety and depression [28,29]. Damage to the DG can cause episodic memory impairment, emotional dysregulation, and deficits in orientation [30]. Here, we demonstrated that intraoperative TI-NIBS targeting the DG can alleviate the core symptoms of POD by substantially increasing neurotransmitter release. Clinical trials of TI-NIBS have reported good tolerability without serious adverse effects. However, larger animal studies and long-term clinical trials are needed to further establish its safety and efficacy, especially for neurocognitive disorders.

Indeed, although many studies have sought to identify a unifying mechanism underlying delirium, it is likely that no single mechanism can explain the full spectrum of causes and manifestations of this heterogeneous syndrome [4,31,32]. In this study, we observed that most neurotransmitters were reduced after surgery, particularly spermine and putrescine, which are biogenic amines involved in regulating homeostasis of excitatory-inhibitory synaptic transmission, neural plasticity, neuroinflammation, and apoptosis. They have been shown to inhibit neuronal apoptosis and inflammation in mice [33,34]. Additionally, along with our findings, recent studies have also demonstrated that spermine administration significantly reverses cognitive impairment induced by lipopolysaccharide (LPS) treatment [35]. Furthermore, we observed that intraoperative TI-NIBS could increase dopamine (DA), serotonin (5-HT), norepinephrine (NE), and glutathione (GSH) concentrations in the hippocampus. Elevated levels of DA, a key regulator of cognitive flexibility and memory consolidation, enhance prefrontal-hippocampal synaptic efficiency by activating D1 receptors, thereby improving executive function [36]. Similarly, heightened NE levels optimize arousal and attention via α2-adrenergic receptor-mediated suppression of locus coeruleus hyperactivity [[37], [38], [39]], while concurrently mitigating neuroinflammation through β-adrenergic modulation of microglial phagocytic activity [40,41]. Increased 5-HT, via hippocampal 5-HT1A receptor signaling, stabilizes emotional responses by dampening stress-induced hyperactivity in the amygdala-hippocampal axis, thereby reducing anxiety-like behaviors [[42], [43], [44], [45]]. Notably, GSH upregulation neutralizes the reactive oxygen species (ROS) generated during perioperative metabolic stress, thus preserving mitochondrial integrity in hippocampal neurons and preventing oxidative damage-induced synaptic dysfunction [46]. These effects may exhibit functional interdependence within individual subjects with POD.

This study has several limitations that warrant consideration. First, while the 3D-AI behavioral analysis system provides high-resolution phenotyping of spontaneous behaviors in mice, we did not subclassify animals undergoing laparotomy with isoflurane anesthesia into distinct POD subtypes (e.g., hyperactive vs. hypoactive). Second, this study focused on contextual memory (evaluated using fear conditioning) and working memory (assessed using the novel object recognition test), but did not address spatial or other memory deficits that are hallmarks of postoperative delirium. Third, although neurotransmitter metabolomics revealed that TI-NIBS selectively induces the hyperfunction of dopaminergic transmission in the hippocampus, the neural substrates of this phenomenon remain unclear.

## 5 Conclusion

Our study provides a comprehensive and nuanced description of behavioral dynamic changes in mice after surgery, offering a data-driven assessment and classification paradigm for individuals with POD. We also demonstrated that intraoperative TI-NIBS prevented POD symptoms, highlighting its potential as a therapeutic tool in clinical postoperative management.

## Data and material availability

All data needed to evaluate the conclusions in the paper are present in the paper and/or the Supplementary Information.

## CRediT authorship contribution statement

**Lei Liu:** Writing – review & editing, Writing – original draft, Methodology, Investigation, Formal analysis, Data curation. **Ye Wang:** Writing – review & editing, Writing – original draft, Methodology, Investigation, Formal analysis, Data curation. **Hui Wang:** Methodology, Formal analysis. **Huimin Wu:** Investigation. **Rou Xue:** Visualization. **Xinxin Zhang:** Software, Conceptualization. **Jingsheng Tang:** Methodology. **Zhouwei Wang:** Methodology. **Tingting Gu:** Investigation. **Dan Wang:** Methodology. **Jiannan Li:** Methodology. **Huiming Li:** Methodology. **Sa Wang:** Methodology, Investigation. **Jinghao Wang:** Investigation. **Yi Zeng:** Supervision. **Haixing Zhong:** Supervision. **Guangchao Zhao:** Writing – review & editing, Supervision, Resources, Project administration, Funding acquisition, Conceptualization. **Hailong Dong:** Writing – review & editing, Supervision, Resources, Project administration, Funding acquisition, Conceptualization.

## Declaration of competing interest

The authors declare that they have no conflicts of interest in this work.

## Appendix Supplementary materials

Image, application 1
