# Supplementary material for: Intraoperative temporal interference (TI)-noninvasive brain stimulation prevents postoperative delirium in mice
Source: Fundam Res. 2025 Aug 16;6(5):3395–407. doi: 10.1016/j.fmre.2025.08.001 (PMC13644489; doi:10.1016/j.fmre.2025.08.001)
Supplement: Supplementary file 1 [file mmc1.docx]

**Supplementary Information**

**Intraoperative Temporal Interference (TI)-Noninvasive Brain Stimulation Prevents Postoperative Delirium in Mice**

By *Lei Liu* et al.

**Supplementary Materials and Methods**

**Open field test**

Prior to the experiment, the apparatus of open field was cleaned with 20% alcohol for eliminating odor. Mice were slightly placed in the center of the paddock, and allowed to move freely for 5 min. The movement trajectory was recorded with the top camera. Total distance traveled in the open field and the time spent in the central area were analyzed by using the ANY-maze animal tracking system software (Stoelting Co., USA).

**Elevated plus-maze test**

The Elevated Plus-Maze (EPM) test was performed following the open field test. The EPM was cleaned and odor removed using 20% alcohol still before the experiment. Mice were gently placed in the central area of the maze and a 5 min video was recorded through the top camera. The ANY-maze animal tracking system software (Stoelting Co., USA) was used to analyze the track of movement trajectory. Time spent in the open arm and number of entering the open arm were calculated for evaluation of anxiety.

**Sucrose preference test**

Mice were acclimatized to a bottle of water and a bottle of 1% sucrose for 24 h. They were then subjected to 24 h of water deprivation, without water or sucrose provided. A sucrose preference test was performed after 24 h of water deprivation. During sucrose preference test, mice were provided with a bottle of 1% sucrose and a bottle of water for a total of 4 h. The positions of the sugar and pure water were replaced at 2 h after the beginning of experiment to prevent positional preference. Water and sucrose consumption were measured at the end of the test. Sucrose preference is the calculation of sucrose consumption as a percentage of total fluid consumption.

**c-Fos immunofluorescence**

Mice were perfused with saline after sacrifice, followed by 4% paraformaldehyde. Brains were extracted, placed in 4% paraformaldehyde for 4 hours of post-perfused, and then transferred to 30% sucrose solution for dehydration for 48 h. Coronal sections of 40 µm thickness were cut using a cryostat microtome (Leica CM1900, Germany). The sections were rinsed 3 times in PBS solution for 10 min each. The brain slices were then permeabilized and blocked for 2 h using 0.3% Triton X-100, 5% donkey serum plus PBS solution. Primary antibody (guinea pig anti-c-Fos 1:800 synaptic Systems, USA) were dissolved in PBS solution supplemented with 2.5% donkey serum and 0.3% triton and mixed well. Brain slices were incubated in diluted primary antibody solution at 4°C for 48 h. After 48 h, brain slices were rinsed three times for 10 min each using PBS and secondary antibody (donkey anti-guinea pig Alexa 488, 1:500, Jackson ImmunoResearch) was dissolved in PBS solution supplemented with 2.5% donkey serum and 0.3% triton and mixed well. Brain slices were placed in the diluted secondary antibody solution and incubated for 2 h at room temperature. Finally, the slices were rinsed 3 times with PBS for 10 min each.

**UHPLC-MS/MS analysis**

Hippocampal tissues were isolated on ice after sacrifice mice and immediately frozen in a -80°C refrigerator. Based on previous method [1]. Benzoyl chloride was used to derivatization carrying out using an ACQUITY Premier (Waters) System, equipped with a Waters ACQUITY UPLC HSS T3 (100 × 2.1 mm, 1.8 μm). SCIEX Analyst Work Station Software (Version 1.6.3) and Biobud-DDF (Version 2.0.3.11) were employed for MRM data processing.

**Short-Long preference (SLP)**

SLP = sin^-1^($\frac{T\text{<1.2s}-T>1.2s}{T<1.2s+T>1.2s}$)

This index serves as a ratio between time long segments and short segments, expressed as an angle. The Short-Long preference ranges from -π/2 to π/2 rad, with -π/2 rad meaning the segments totally longer than 1.2s, π/2 rad meaning that the segments totally shorter than 1.2s, and 0 rad meaning equal preference for long and short segments [2].

**Novel object recognition**

We used a new object recognition experiment to test the working memory of mice, and the mice were placed in the test room 1 hour before the test to acclimatize. The experimental process is divided into 2 steps, the training phase and the test phase, in the training phase, the mouse is gently placed in the center of the open field, two similar identical objects are placed on the side of the open field to allow the mouse to explore freely for 10 minutes, and then the mouse is returned to the home cage. The testing phase begins after 1 h, one of the two similar objects is swapped out for a novel one, and the mouse is placed in the center of the open field for 5 min of exploration. Use ANY-maze to record and analyze the time spent exploring novel and old objects during the testing phase.

**Supplementary Table 1**

**Definition of the behaviors (ethogram) for manual labeling**

| **Category** | **Behavior** | **Definition** |
| --- | --- | --- |
| Exploration | Rising | The mouse rises from four legs on the ground to steadily stand on its hind legs. |
|  | Sniffing | The mouse investigates environment with the nose held in the air or contacts the environment with nose closely. |
|  | Up stretching | The mouse stands on its hind legs while the front part of body stretches back and forth. |
|  | Scrunch sniffing | The mouse arches its back and investigates environment with the nose held in the air or contacts the environment with nose closely. |
| Numbing | Curling | The mouse buried head into its abdomens in place. |
|  | Immobility | The mouse remained motionless in place |
| Locomotion | Running | The mouse locomotes with relatively high speed. |
|  | Trotting | The mouse locomotes at a slow and intermittent pace. |
|  | Walking | The mouse locomotes with relatively low speed. |
|  | Turning | The mouse turns body to left or right while walking. |
| Emotion | Jumping | The mouse jumped up off the ground. |
|  | Grooming | The mouse licks its fur, grooms with the forepaws, or scratches with any limb. |
|  | Hunching | The mouse stands on its hind legs while the back is bent. |
| Gait change | Head shaking | The mouse shook its head from side to side |
|  | Scrunch move | The mouse arches its back and locomotes with relatively low speed. |

**Reference**

[1] .	Y. Wang, Y. Zhang, K. Wang et al., Esketamine increases neurotransmitter releases but simplifies neurotransmitter networks in mouse prefrontal cortex: Journal of neurophysiology. 127 (2022) 586-595.

[2] .	S. Cao, Y. Wu, Z. Gao et al., Automated phenotyping of postoperative delirium-like behaviour in mice reveals the therapeutic efficacy of dexmedetomidine: Communications biology. 6 (2023) 807.

**Supplementary Figures and Figure legends**

**Fig. S1. Motional Bradykinesia and Hypo-flexibility after Surgery**

(a) Comparison between two groups after normalization of 38 kinematic parameters (n = 8-9). (b) Differences in the distribution of behavioral segment lengths after surgical modification (n = 8-9). (c) Left: Probability in preference for behavioral segment lengths. Right: Statistical analysis of differences in preference for behavioral segment lengths (n = 8-9). (d) Differences in the number of behavioral transitions (n = 8-9). Statistics: two-way ANOVA followed by Holm-Sidak post *hoc* multiple comparisons test in a, b**,** two-sided Kolmogorov-Smirnov test in **c**, two-sided unpaired t-test in d**.** ** *p* < 0.01, **** *p* < 0.0001.

**Fig. S2. 3D-AI behavior analysis can capture more details about the animals than ANY-Maze.**

(a) ANY-maze 7.4 behavioral analysis depicted the movement trajectories of the sham group and the surgery group. (b) Movement trajectories depicted by 3D-AI-assisted behavioral analysis, the vertical axis shows the characteristics of the trajectory over time, with yellow representing faster motion and blue representing slower motion. (c-e) Comparison of the total distance (sham, *p* = 0.8338; surgery, *p* = 0.9632), maximum speed (sham, *p* = 0.7969; surgery, *p* = 0.7969), and time spend in center (sham, *p* = 0.9651; surgery, *p* = 0.9937) of the two analysis systems in the open field test (n = 8-9, statistics: two-way ANOVA followed by Holm-Sidak post *hoc* multiple comparisons test).

**Fig. S3. Emotion and Contextual Memory Change after Surgery**

(a) Typical heat map of the position of mice within the open field. (b) Difference of total distance and time in the center zone (n = 8-9) in the open field test. (c) Typical trajectory of mice in an elevated plus maze. (d) Difference of the number to the open arm and the time in the open arm (n = 8-9) of the elevated plus maze. (e) Comparison of the sucrose preference between the shams and surgeries (n = 8-9). (f) Difference of the freezing time ratio during the 5 sounds stimuli of fear condition training between the shams and surgeries (n = 8-9). (g) Difference in the percentage of freezing between the two groups during the contextual memory test (n = 8-9). Statistics: two-sided unpaired t-test in b, d, e, g, two-way ANOVA followed by Holm-Sidak post *hoc* multiple comparisons test in f. * *p* < 0.05, ** *p* < 0.01, *** *p* < 0.001.

**Fig. S4. The expression of c-Fos in the stimulated side hippocampus increased after TI-NIBS intervention**

(a) Typical image of c-Fos immunofluorescence staining of the dorsal hippocampus. (b) Statistical plot of c-Fos positive cell counts in the dorsal hippocampus (n = 6, no TI-NIBS 38.17 ± 3.167 vs. TI-NIBS 52.50 ± 1.607, statistics: two-sided paired t-test, ** *p* = 0.0012).

**Fig. S5. Intraoperative TI-NIBS Alleviates Bradykinesia and Hypo-flexibility after Surgery**

(a) Comparison between two groups after normalization of 38 kinematic parameters (n = 8). (b) Differences in the distribution of behavioral segment lengths after surgical modification and TI-NIBS (n = 8-9). (c) Left: Probability in preference for behavioral segment lengths after surgical modeling. Right: Statistical analysis of differences in preference for behavioral segment lengths after surgical modeling (n = 8-9). (d) Differences in the number of behavioral transitions after surgical modification (n = 8-9). Statistics: two-way ANOVA followed by Holm-Sidak post *hoc* multiple comparisons test in a, b, one-way ANOVA followed by Holm-Sidak post *hoc* multiple comparisons test in c, d. * *p* < 0.05, **** *p* < 0.0001.

**Fig. S6. Intraoperative TI-NIBS Alleviates Anxiety and Memory Deficit after Surgery**

(a) Comparison of total distance in the open field test. (b) Difference of total distance and time in the center zone in the open field after TI-NIBS (n = 8-9). (c) Typical trajectory of mice in an elevated plus maze. (d) Difference of the number to the open arm and the time in the open arm (n = 8-9). (e) Comparison of the sucrose preference between the surgery no-TI group and surgery TI group (n = 6-9). (f) Difference of the freezing time ratio during the 5 sound stimuli of fear condition training between the surgery-no TI group and surgery TI group (n = 6-9). (g) Difference in the percentage of freezing (n = 6-9). (h) Left: the typical graph of the novel object recognition experiment; right: the statistical results of the new object exploration time (n = 10). Statistics: one-way ANOVA followed by Holm-Sidak post *hoc* multiple comparisons test in b, d, e, two-way ANOVA followed by Holm-Sidak post *hoc* multiple comparisons test in f, g, h. * *p* < 0.05, ** *p* < 0.01, *** *p* < 0.001, **** *p* < 0.0001.
